# Epidemiological considerations on HIV infection in Constanța region

**DOI:** 10.1186/1471-2334-14-S7-P30

**Published:** 2014-10-15

**Authors:** Iulia Gabriela Șerban, Sorin Rugină

**Affiliations:** 1 Regional HIV/AIDS Center Constanța, Romania; 2 Faculty of Medicine, “Ovidius” University, Constanța, Romania

## Background

Lack of available epidemiological studies and circulation of unverified and unsubstantiated scientific information contributed to the uncertainty regarding the onset, causes and epidemiological developments in Romania. This paper aims to formulate epidemiological considerations on evolution of HIV infection based on a retrospective and prospective epidemiological analysis of 2 groups (A and B) of patients from Constanța, period 1987-2013.

## Methods

We processed the existing database, records of the consultations, surveys on assessing health status, pathological, laboratory data, clinical observation on multicriterial sheets, the register of deaths and pathological examinations, presented in 3 studies: epidemiological retrospective description from 1987 to 1993, prospective in period 2008 – 2012 and during the period January – 30 June 2013.

## Results

Group A (acute evolution – from the 1987 to 1989 cohort, the natural history of the disease), survival rate: 18-24 months initially, then 4-5 years, received only palliative care and medical assistance, causes of death: opportunistic infections and high mortality.

Group B contains two segments: the 1987-1989 cohort and patients infected by other routes: heterosexual, IDU, MSM, MTCT), with chronic evolution between 2008 – June 30, 2013, patients undergoing antiretroviral therapy 10-11 years (2008), 12-13 (2008), -18 years (2013), both derived from cohort and newly detected, average survival 10 to 11 years, reduced mortality, predominantly through TB or pneumocystosis, polyexperimented patients, exhausted immunological or non-adherent status. There are long-term survivors (slow progressors and non- progressors), which completed, part of them, the natural history of the first group, provided they did not need treatment, because of a less influenced immunological status.

There are four models of evolution: group A – acute and over-acute evolution – fast progressors (mortality rate increased, duration of survival of 4-5 years), group B – chronic evolution – late presenters, slow progressors, non-progressors – the 3 new "patterns" are correlated with an increase in average life expectancy to 10.5-11 years, due to, in the overwhelming majority, the therapy and the natural history). We estimate an evolution of the HIV-AIDS epidemic in Constanța County, on an average of 7-9 years, in the same parameters, which were characterized by the current study.

## Conclusion

These results generate special concern in future epidemiological studies in depth to give the actual size of the phenomenon of HIV-AIDS in Constanța, and in Romania.

